# Digital strain in the workplace: the role of instrumental social platform use on employee emotional exhaustion

**DOI:** 10.3389/fsoc.2026.1878074

**Published:** 2026-08-28

**Authors:** Jun Zhou, Mengni Wei, Jinhao Huang, Qimeng Dai

**Affiliations:** 1 School of Business, Changzhou University, Changzhou, China; 2 School of Innovation Strategy and Talent Development, Changzhou University, Changzhou, China; 3 School of Law, Wenzhou University, Wenzhou, China

**Keywords:** digital fatigue, digital literacy, emotional exhaustion, instrumental use of social platforms, work-family conflict

## Abstract

**Purpose:**

As digitalization increasingly permeates work environments, employees' persistent use of social platforms for task management, collaboration, and information sharing (i. e., instrumental use) has become widespread. However, its potential negative impact on mental health remains under-examined. To explore this phenomenon, this study constructs a chained path model—“social tool use → digital fatigue → work-family conflict → emotional exhaustion”—based on resource conservation theory and boundary theory, while examining the moderating role of digital literacy.

**Design/methodology/approach:**

Through empirical research, this study constructed and validated a chained mediation model. Structural equation modeling and other methods were employed to examine the pathways from “social tool use → digital fatigue → work-family conflict → emotional exhaustion” and the moderating effect of digital literacy.

**Findings:**

(1) Instrumental use of social platforms significantly positively associated with emotional exhaustion; (2) Digital fatigue and work-family conflict not only mediate this relationship individually but also form a chained mediating association; and (3) Digital literacy effectively mitigates the negative impact of social tool use on digital fatigue.

**Originality/value:**

This research deepens and expands the understanding of the formation mechanisms of stressors in digital work environments, helping enterprises navigate the dual effects of social platform tools. It provides “mitigation” strategies for companies that have already deployed such tools, while also offering decision-making and implementation guidance for those still observing.

## 1 Introduction

The capacity of digital technology can afford both profound benefits and substantial harm (Recker et al., 2025). Enterprises are increasingly mandating constant connectivity via social platforms such as WeChat or Facebook. Although this integration has streamlined communication flows and enhanced productivity (Fazil et al., 2024), the commodification of these once-recreational tools has paradoxically turned them into significant stressors for employees. What was ostensibly intended to “reduce workload” has revealed a contradictory reality in practice: the mandate for constant connectivity erodes work-life boundaries, compelling employees to respond to messages after hours and during leave. Consequently, this leads to a covert extension of working hours, accompanied by a marked increase in psychological strain and burnout (Demerouti and Adaloudis, 2024).

According to statistics, Chinese residents spend less than 3.5 h working on average each day but more than 5.5 h online, which reflects the blurred boundary between work and life. The popularity of remote work and online collaboration tools has made many people feel that “being online means being at work.” In the past 2 years, some employees have even had legal disputes with employers because they had to keep working long hours through social software after returning home, and the courts ruled that employers should pay for such “invisible overtime.”

The widespread adoption of social media for task-oriented, instrumental purposes has raised concerns about its potential toll on employees' mental health. According to the Conservation of Resources (COR) theory, such high-intensity, work-related use demands sustained cognitive and emotional investment, depleting employees' limited resources and hindering recovery after work (Schlachter et al., 2025; Demerouti and Adaloudis, 2024). A key consequence of prolonged instrumental use is digital fatigue—a state of psychological and physical depletion induced by sustained digital engagement, characterized by attentional decline and emotional strain. However, existing research has rarely unpacked the mediating mechanisms through which instrumental social media use leads to emotional exhaustion, and even less attention has been paid to the sequential mediating pathways linking these constructs.

Building on COR theory and boundary theory, we propose that instrumental social media use triggers a sequential process: first, it depletes employees' cognitive and emotional resources, inducing digital fatigue; in turn, this fatigue weakens employees' ability to maintain boundaries between work and family, exacerbating work–family conflict; ultimately, this sustained resource drain and role conflict culminate in emotional exhaustion. This chain mediation model offers a more nuanced explanation of how instrumental use translates into adverse health outcomes, moving beyond simple direct-effect or single-mediator accounts.

**Figure 1:**
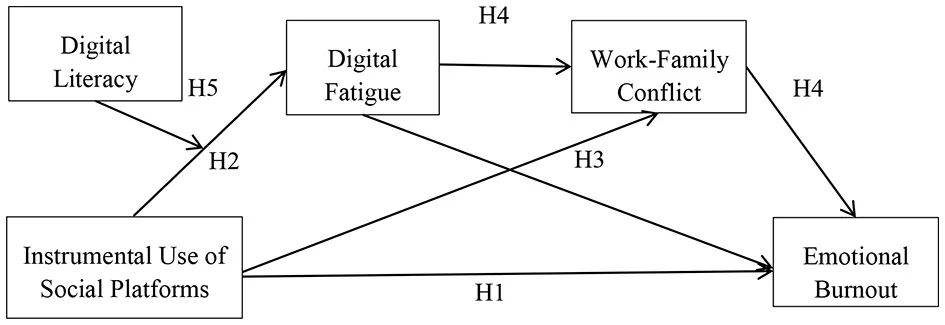
Concept model.

To test this proposition, this study develops and empirically examines a sequential mediation model (Figure 1). The theoretical contributions are twofold: First, we shift the focus from general social media usage frequency to instrumental use, revealing its distinctive impact on employee wellbeing through a cascading resource-depletion process. Second, by integrating digital fatigue and work–family conflict as serial mediators, we uncover the stepwise mechanism through which work-related social media use ultimately leads to emotional exhaustion, offering a more complete picture of the underlying process.

The structure of this paper is as follows: first, we review previous studies and real-world issues to develop the research hypotheses; second, we test the hypotheses and the moderating role of digital literacy using multiple mediation path analysis; finally, based on the results, we discuss theoretical implications and practical suggestions, providing insights for organizations to promote employee mental health and reduce emotional exhaustion in digital office environments.

## 2 Theoretical background and hypotheses development

### 2.1 The definition of instrumental use of social media platforms and its impact on emotional exhaustion

Regarding the definition of instrumental use of enterprise social media, the academic community has not yet reached a unified terminology. Various expressions exist, including “Enterprise 2.0” (McAfee, 2006), enterprise social media (Kaplan and Haenlein, 2010), enterprise social software platforms, and internal enterprise social networks. Although these concepts differ slightly in terminology and emphasis, they share a common underlying meaning: organizational members use social media platforms as functional tools to achieve work objectives and facilitate organizational communication and collaboration. Based on this understanding, instrumental use of social media should be distinguished from general social media use. General social media use refers to individuals'engagement with social media platforms driven by personal needs, such as entertainment, interpersonal interaction, emotional expression, or information acquisition (Kircaburun et al., 2020). Its primary purpose is to satisfy users'personal social or recreational needs. In contrast, instrumental use emphasizes the task-oriented functions of social media within organizational work contexts, where employees utilize these platforms to accomplish work tasks, share information, coordinate activities, and facilitate collaboration (Luqman et al., 2021; Wu et al., 2021). Therefore, the distinction between the two does not lie in the type of platform being used (e.g., WeChat, Facebook, DingTalk, or organization-developed systems), nor in the frequency of use, but rather in the user's purpose and the application context (Cao et al., 2016). When employees rely on social media in workplace settings for task-related communication, knowledge exchange, relationship coordination, and team collaboration, such use can be regarded as instrumental social media use.

Existing studies suggest that the effects of instrumental use of social media are dual in nature. On the positive side, Rahman et al. (2025) found that enterprise social networks can facilitate knowledge sharing and improve organizational information exchange by strengthening employees' connections and collaboration; Raub et al. (2021) confirmed that task-oriented and social-oriented use can improve performance. However, the negative effects should not be overlooked: Wright and Cropanzano (1998) noted that emotional exhaustion can trigger a vicious cycle of “performance decline–increased anxiety.” As a core component of burnout, it often coexists with depersonalization and reduced sense of accomplishment, serving as a key indicator of workplace stress (Demerouti, 2024). Islam et al. (2021) reported that excessive use may lead to information overload, privacy concerns, and social pressure, resulting in cognitive fatigue and burnout; Üztemur et al. (2025) found that high-frequency use is positively associated with work-related emotional exhaustion. These findings indicate that when promoting instrumental use of social media, organizations need to balance its potential for efficiency gains with its possible risks.

To further explain its possible negative outcomes, Conservation of Resources (COR) theory (Hobfoll, 1989; Halbesleben et al., 2014) can be applied. The core idea of this theory is that individuals strive to gain, keep, and protect their resources, and stress occurs when these resources are threatened or lost. While the instrumental use of social media can improve communication efficiency, it may also lead to resource depletion. When employees frequently receive and process instant messages, their attention, energy, and time—resources that are limited—are continuously consumed (Angelini, 2023). Such overuse prevents them from focusing on core tasks, increases psychological pressure (Song and Kim, 2026). Especially during nonwork hours, social media use extends work boundaries (Fang et al., 2024), making it harder for employees to rest properly, which further contributes to fatigue and emotional exhaustion (Ji and Jan, 2024). Therefore, we propose:

*Hypothesis 1: The instrumental use of social media is positively associated with employees' emotional exhaustion*.

### 2.2 The mediating role of digital fatigue and work–family conflict

With the widespread use of digital technologies and social media tools, work fatigue has increasingly appeared in the form of digital fatigue. Digital fatigue refers to the cognitive overload and psychological exhaustion caused by excessive reliance on digital technologies and continuous online engagement (Posnock, 2013). Prior studies have found that in online education, students' constant use of technologies—such as participating in virtual classes, video conferencing, and digital communication—can also lead to mental fatigue (Prasetyo et al., 2025). For employees, handling work-related information and using communication technologies after hours are key sources of digital fatigue. Engaging in work through digital tools during nonwork hours increases psychological detachment from work, thereby elevating levels of digital fatigue (Lee et al., 2021).

Previous studies have shown that digital fatigue has adverse effects on employees' psychological wellbeing and job performance. Thomée et al. (2007) found that digital fatigue undermines employees' physical and mental health, reduces sleep quality, and weakens attention at work; once emotional strain reaches a critical threshold, employees are likely to fall into full emotional exhaustion (Beauchamp Legault et al., 2025). Similarly, Supriyadi et al. (2025) demonstrated that digital fatigue caused by excessive use of digital communication tools not only reduces employees' work efficiency but also diminishes their overall wellbeing.

This study argues that the instrumental use of social media platforms increases employees' digital fatigue, which in turn leads to emotional exhaustion. From the perspective of COR theory, when employees frequently process instant messages, engage in cross-platform information sharing, and manage multitasking collaboration, their limited resources—such as attention, time, and emotional regulation—are continuously depleted. The lack of opportunities for recovery creates resource imbalance (Demerouti, 2025). In this process, resource depletion due to prolonged exposure to digital tools constitutes the essence of digital fatigue. This fatigue, triggered by resource loss, further generates chronic stress and emotional exhaustion (Thomée et al., 2007; Supriyadi et al., 2025). Thus, we hypothesize as follows:

*Hypothesis 2A: The instrumental use of social media is positively associated with digital fatigue*.

*Hypothesis 2B: Digital fatigue mediates the relationship between the instrumental use of social media and emotional exhaustion*.

Building on the mechanism of digital fatigue, it is also important to examine how the instrumental use of social media affects employees' role boundaries. While digital fatigue reflects the ongoing depletion of resources at the individual level, work–family conflict reveals the competition and tension of resources at the role level. Work–family conflict refers to the strain experienced when expectations and responsibilities from work and family roles are incompatible, including both work interfering with family and family interfering with work (Greenhaus and Beutell, 1985). Previous research has often relied on boundary theory to explain the underlying mechanisms of work–family conflict (Kossek and Lautsch, 2008). Boundary theory says that people set mental, time, and space boundaries to separate work from family and other life areas. When technology use is forced or intrusive, it can take away employees' sense of control over these boundaries (Hsu et al., 2024). How easily boundaries are crossed and how flexible they are can directly affect role conflicts, happiness, and work performance (Rothbard, 1999). In the digital era, social media and mobile work technologies have significantly increased boundary permeability, making it more likely for work to intrude into the family domain and thereby raising the risk of work–family conflict. Since instrumental use of social media often places employees in an “always online” state, work-related tasks frequently spill over into nonwork hours, consuming the time and energy needed to fulfill family responsibilities. This prolonged resource depletion not only reduces employees' wellbeing but also undermines their emotional regulation capacity, ultimately resulting in emotional exhaustion. Hence, the following hypothesis is proposed.

*Hypothesis 3A: The instrumental use of social media is positively associated with work*–*family conflict*.

*Hypothesis 3B: Work*–*family conflict mediates the relationship between the instrumental use of social media and emotional exhaustion*.

Furthermore, digital fatigue is not only a manifestation of resource depletion at the individual level but may also intensify cross-role resource competition, thereby influencing the emergence of work–family conflict. Specifically, when employees experience cognitive overload and psychological fatigue due to frequent instrumental use of social media, their available attention, emotional regulation capacity, and time resources are significantly reduced (Posnock, 2013; Lee et al., 2021). Under such resource-constrained conditions, employees find it more difficult to manage the demands of both work and family roles, which increases the likelihood of role conflict (Greenhaus and Beutell, 1985). Prior research has demonstrated that various forms of work-related fatigue can intensify conflicts between different life roles (Huang et al., 2023). More specifically, when prolonged use of social media tools induces digital fatigue, employees' cognitive and emotional resources are substantially depleted (Madan et al., 2025), making it harder to maintain balance between work and family roles and thus increasing the probability of work–family conflict (Greenhaus and Beutell, 1985). Digital fatigue, as a form of psychological resource depletion, indirectly exacerbates cross-role resource competition by weakening employees' control over role boundaries, creating a chain effect from the individual level to the role level. This leads us to the following hypothesis:

*Hypothesis 4: Digital fatigue and work*–*family conflict jointly play a chain mediating role in the relationship between the instrumental use of social media and emotional exhaustion*.

### 2.3 The moderating role of digital literacy

In the context of digitalized work and the widespread adoption of social media platforms, employees are required to constantly process large amounts of information and use multiple digital tools to accomplish work tasks. This places higher demands on their cognitive capacity, information-processing skills, and self-management abilities. Therefore, digital literacy—defined as individuals' comprehensive ability to access, understand, evaluate, and use information effectively in digital environments—has become an important concept in examining how employees cope with stress and negative outcomes in digital workplaces (Eshet, 2004; Havrilova and Topolnik, 2017). Digital literacy generally consists of three dimensions: skills, strategies, and metacognitive abilities (Eshet, 2004; Havrilova and Topolnik, 2017). Specifically, it includes not only the technical skills to operate digital tools and platforms, but also the strategies to effectively filter and apply information, and the metacognitive ability to manage and regulate one's cognition and emotions. By integrating these capabilities, digital literacy enhances employees' efficiency, resource utilization, and stress regulation in digital environments (Pool, 1997).

Existing research shows that digital literacy is not only a key skill for individuals to survive and develop in the digital era, but also an important personal resource that can help buffer the negative effects of technology use (Wu et al., 2026). For example, a high level of digital literacy improves employees' ability to filter information and communicate efficiently, making them more effective when using digital platforms and social tools, thereby reducing cognitive overload and digital fatigue (Sualeh Khattak et al., 2024). In addition, employees with strong metacognitive abilities can actively regulate themselves and restore resources when experiencing stress or fatigue, which reduces the risks of emotional exhaustion and work–family conflict (Jiang et al., 2025). By contrast, employees with lower digital literacy may struggle with information filtering, tool usage, and task management, becoming easily overwhelmed by large amounts of information. This accelerates resource depletion and increases digital fatigue, emotional exhaustion, and cross-role conflict. Thus, digital literacy, as an individual resource, plays an important moderating role in coping with stress and negative outcomes in digital work environments.

According to the COR theory, individuals under stress will strive to protect and accumulate their limited resources (Hobfoll, 1989). In digital workplaces, higher levels of digital literacy enable employees to use tools more effectively, filter information more quickly, and manage their time and attention resources more efficiently, thereby reducing the resource loss and psychological fatigue caused by instrumental use of social media. When employees have high digital literacy, they can better handle information overload and multitasking demands, buffering the resource consumption caused by instrumental use and reducing digital fatigue (Moughal et al., 2023). Conversely, employees with lower levels of digital literacy are more likely to feel overwhelmed, experience accelerated resource depletion, and suffer from higher levels of digital fatigue. Thus, our final hypothesis is as follows.

*Hypothesis 5: Digital literacy negatively moderates the relationship between instrumental use of social media and digital fatigue, such that the positive association between instrumental use of social media and digital fatigue is weaker among employees with higher levels of digital literacy*.

### 2.4 Research model

Based on the above analysis, this study proposes the conceptual framework illustrated in Figure 1. This framework provides the theoretical foundation for the subsequent research design, the selection of measurement instruments, and the empirical analysis.

## 3 Method

### 3.1 Research setting and sample

This study aims to investigate and analyze users across several industries in China who utilize social media platforms as tools. The participants were employees from several industries in the Yangtze River Delta region of China, including Jiangsu, Zhejiang, and Shanghai. Since this study focuses on employees' experiences with instrumental social media use in the workplace, we recruited participants who had practical experience using social media platforms for work-related activities. Specifically, we contacted human resource managers from several enterprises and invited employees who had worked in their current positions for more than 3 months to participate in the survey. A combination of purposive and convenience sampling methods was adopted. The purposive sampling approach was used to ensure that participants met the research requirements regarding workplace experience and social media use, while convenience sampling was applied in the recruitment process through accessible enterprise contacts. During the questionnaire survey, participants were asked to recall their social media usage experiences over the previous week and report their current feelings of digital fatigue and related psychological states. The questionnaire was distributed both in person and online, with 432 copies issued and 403 returned. After excluding incomplete responses and those exhibiting obvious patterns, 381 valid questionnaires were obtained (188 males, 193 females), yielding a valid response rate of 88.19%. Age distribution: 21% aged 25 and under; 26.2% aged 26–30; 24.2% aged 31–40; 22.3% aged 41–50; 6.3% aged 50 and over. Educational attainment: 9.2% held master's degrees or higher; 27.3% held bachelor's degrees; 39.4% held associate degrees, and 24.1% had high school diplomas or below. By job level, 43% were frontline employees, 21% were frontline managers, 26% were middle managers, and 10% were senior managers. Regarding years of work experience, 36.5% had 1–5 years, 34.1% had 6–10 years, 11–20 years accounted for 21.3%, and 21–30 years accounted for 8.1%. Regarding daily social media usage time, less than 2 h accounted for 15%, 2–4 h accounted for 32.5%, 4–6 h accounted for 22.6%, 6–8 h accounted for 18.6%, and more than 8 h accounted for 11.3%.

### 3.2 Variable measurement

The core variables in this study include instrumental use of social media, digital fatigue, emotional exhaustion, work-family conflict, and digital literacy. To ensure measurement reliability and validity, established scales widely used internationally were adopted. For English-language scales, items underwent a two-way back-translation process. Final scales were determined through a review of domestic and international literature combined with expert input. All core variables were measured using a five-point Likert scale ranging from “1–Strongly Disagree” to “5–Strongly Agree.”

#### 3.2.1 Instrumental use of social media

This variable employs the scale revised by Cao et al. (2016). The work-based corporate social platform usage scale focus on the instrumental functions of social media, emphasizing the use of social media to facilitate work communication, information sharing, and task collaboration, thereby distinguishing it from general social media use. This variable consists of five items, with representative examples including: “I use social platform tools to set up groups for discussing work project information with colleagues” and “I use social platform tools for storing and sharing corporate document information.” The scale's Cronbach's α is 0.871.

#### 3.2.2 Digital fatigue

This variable references Ayyagari et al.'s (2011) digital fatigue measurement scale. The scale comprises four items, with representative examples including: “I feel extremely exhausted when using digital devices for work” and “Using digital devices for work makes me feel fatigued.” The scale's Cronbach's α was 0.871.

#### 3.2.3 Emotional exhaustion

Adapted from Schaufeli (1996)'s emotional exhaustion scale. This scale comprises five items, with representative items including: “I feel unmotivated and exhausted when facing work” and “I feel overly fatigued after completing daily work.” The scale's Cronbach's α is 0.883.

#### 3.2.4 Work-family conflict

Referenced the work-family conflict scale developed by Carlson and Kacmar (2000). This scale comprises five items, with representative items including: “My job demands so much time that I have little time left for my family” and “When I get home from work, I often feel too tired to participate in family activities.” The scale's Cronbach's α was 0.850.

#### 3.2.5 Digital literacy

Measured using a scale adapted from Nikou et al.'s (2022), comprising eight items. Representative items include: “I can use common office software” and “I can independently resolve problems encountered during work.” The scale's Cronbach's α was 0.895.

#### 3.2.6 Control variables

Prior research in the context of enterprise digital technologies has commonly included demographic characteristics, such as gender, age, education level, and job level, as control variables because these factors may influence employees' technology usage patterns, digital capabilities, work demands, and psychological responses to technology (Tarafdar et al., 2007; Song and Kim, 2026). Therefore, this study controlled for participants' age, gender, educational background, and job level to rule out the potential effects of these factors on the research findings.

### 3.3 Data analysis

#### 3.3.1 Common method bias testing

Given that questionnaire data were collected from a single source, harman's one-factor test was a potential concern. Prior to empirical analysis, further testing was required to determine whether homoscedasticity was severe. This study employed Harman's one-factor test for verification. Exploratory factor analysis results for all measurement items revealed that the maximum factor variance explained was 39.854% under unrotated conditions—below the 40% threshold—indicating that homoscedasticity was not a significant concern.

#### 3.3.2 Reliability testing

Cronbach's α coefficient was used to measure the reliability of each variable scale. The Cronbach's alpha coefficients for all variables in the questionnaire exceeded 0.7, indicating that the questionnaire possesses good reliability (Table 1).

**Table 1:** Reliability analysis.

Variable	Number of measurable variables	Cronbach's α coefficient
Instrumental use of social media	5	0.871
Digital fatigue	4	0.871
Emotional exhaustion	5	0.883
Work-family conflict	5	0.850
Digital literacy	8	0.895
Overall	27	0.922

#### 3.3.3 Confirmatory factor analysis and common method bias testing

As shown in Table 2, the five-factor model demonstrated significantly superior fit indices (χ^2^/df = 1.492, CFI = 0.977, TLI = 0.974, RMSEA = 0.036) compared to alternative models, while fully meeting criteria for good fit (χ^2^/df < 3, CFI > 0.95, TLI > 0.95, RMSEA < 0.05). This indicates that the five core variables in this study possess good discriminant validity and can be treated as independent constructs for research. As the degree of variable merging increased, the model fit indices progressively deteriorated. The four-factor model (merging work-family conflict and digital literacy) exhibited fit indices (χ^2^/df = 3.550, CFI = 0.920, TLI = 0.907, RMSEA = 0.082) exceeding acceptable levels, significantly underperforming the five-factor model. The three-factor model's fit indices (χ^2^/df = 5.194, CFI = 0.905, TLI = 0.887, RMSEA = 0.105) no longer met the criteria for good fit. The two-factor and single-factor models exhibited even poorer fit indices, further validating the conceptual differentiation proposed in this study.

**Table 2:** Confirmatory factor analysis.

Model	Factor	X^2^/df	IFI	TLI	RMSEA
Five-factor	A,B,C,D,E	1.492	0.977	0.974	0.036
Four-factor	A,B,C,D+E	3.550	0.920	0.907	0.082
Three-factor	A,B,C+D+E	5.194	0.905	0.887	0.105
Tow-Factor	A,B+C+D+E	8.493	0.886	0.858	0.140
Single-factor	A+B+C+D+E	7.510	0.681	0.655	0.131

N = 381.

+ indicates two factors combined into one; A (Instrumental use of social media); B (Digital Fatigue); C (Emotional Exhaustion); D (Work-Family Conflict); E (Digital Literacy).

#### 3.3.4 Descriptive statistics and correlation analysis

According to descriptive statistics (see Table 3), the mean score for instrumental use of social media was 3.694 ± 0.909 (range 1–5), indicating respondents rated instrumental use at a moderate level. Digital fatigue scores were relatively high, averaging 3.555 ± 0.981, potentially reflecting existing digital fatigue from current instrumental use of social media. The mean work-family conflict score was 3.486 ± 0.757, indicating that most users perceive a relatively high level of work-family conflict stemming from instrumental use of social media. The mean emotional exhaustion score was 3.529 ± 0.945, suggesting respondents generally exhibit a high level of emotional exhaustion toward instrumental use of social media. The mean digital literacy score was 3.406 ± 0.753, indicating the sample group possesses moderate digital literacy. Regarding control variables, males constituted approximately 49.3% (coded as 1, mean 1.51), with the sample's average age being 2.67 ± 1.213 (categorical variable). The average education level was 2.22 ± 0.915 (categorical variable), and the average job level was 2.03 ± 1.045 (categorical variable). This indicates that the sample exhibits good representativeness and diversity in demographic characteristics, providing a solid foundation for the generalizability of the research findings.

**Table 3:** Descriptive statistics.

Variable	Sample size	Mean	Standard deviation	Minimum value	Maximum value
Gender	381	1.51	0.501	1	2
Age	381	2.67	1.213	1	5
Education level	381	2.22	0.915	1	4
Job level	381	2.03	1.045	1	4
Instrumental use of social media	381	3.694	0.909	1.2	5
Digital fatigue	381	3.555	0.981	1.25	5
Emotional exhaustion	381	3.529	0.945	1.2	5
Work-family conflict	381	3.486	0.757	1.4	4.6
Digital literacy	381	3.406	0.753	1.25	5

Correlation analysis was conducted for each variable, with results presented in Table 4. Findings indicate significant correlations between instrumental use of social media and emotional exhaustion (*r* = 0.689, *p* < 0.01), instrumental use of social media and digital fatigue (*r* = 0.675, *p* < 0.01), digital fatigue and emotional exhaustion (*r* = 0.886, *p* < 0.01), instrumental use of social media and work-family conflict (*r* = 0.728, *p* < 0.01), and work-family conflict and emotional exhaustion (*r* = 0.853, *p* < 0.01). *p* < 0.01), tool-oriented use of social media and work-family conflict (*r* = 0.728, *p* < 0.01), work-family conflict and emotional exhaustion (*r* = 0.853, *p* < 0.01), digital fatigue and work-family conflict (*r* = 0.894, *p* < 0.01). These correlations were all positive and statistically significant, indicating close relationships among the variables and providing preliminary support for subsequent analyses.

**Table 4:** Correlation analysis of variables (*N* = 381).

Variable	1	2	3	4	5	6	7	8	9
1. Gender	1								
2. Age	−0.059	1							
3. Educational Attainment	−0.101^*^	0.525^**^	1						
4. Job Level	−0.068	0.546^**^	0.406^**^	1					
5. Instrumental use of social media	−0.035	0.227^**^	0.266^**^	0.211^**^	1				
6. Digital Fatigue	−0.072	0.279^**^	0.328^**^	0.187^**^	0.675^**^	1			
7. Emotional Exhaustion	−0.07	0.208^**^	0.289^**^	0.102^*^	0.689^**^	0.886^**^	1		
8. Work-Family Conflict	−0.095	0.286^**^	0.341^**^	0.205^**^	0.728^**^	0.894^**^	0.853^**^	1	
9. Digital Literacy	0.011	−0.144^**^	−0.087	−0.075	0.331^**^	−0.275^**^	−0.079	−0.131^*^	1
M	1.51	2.67	2.22	2.03	3.694	3.555	3.529	3.486	3.347
SD	0.501	1.213	0.915	1.045	0.909	0.981	0.945	0.759	0.753

^*^p < 0.05; ^**^p < 0.01.

Given the high correlation coefficient between digital fatigue and work-family conflict, additional analyses were conducted using a competing model comparison approach to examine their discriminant validity. The results showed that the two-factor model (χ^2^ = 42.441, df = 26, χ^2^/df = 1.632, CFI = 0.992, TLI = 0.989, RMSEA = 0.041) demonstrated a better fit than the one-factor model (χ^2^ = 53.968, df = 27, χ^2^/df = 1.999, CFI = 0.987, TLI = 0.983, RMSEA = 0.051). Moreover, the chi-square difference test indicated that the two models differed significantly (Δχ^2^ = 11.527, Δdf = 1, *p* < 0.001). Therefore, digital fatigue and work-family conflict demonstrate satisfactory discriminant validity.

#### 3.3.5 Testing for main effects and mediating effects

This study employed hierarchical regression to test main effects and mediating effects, with results presented in Table 5. First, the main effect validation revealed that Model 5 represents the regression model of control variables on emotional exhaustion. Model 6 adds instrumental use of social media to Model 5, indicating that instrumental use of social media is significantly positively associated with emotional exhaustion (*β* = 0.667, *p* < 0.001), thus validating the H1 hypothesis. Second, the mediating effects of digital fatigue and work-family conflict were examined. Models 2 and 4 separately tested the influence of instrumental use of social media on digital fatigue and work-family conflict, respectively. Results indicate that instrumental use of social media significantly and positively affects digital fatigue (*β* = 0.630, *p* < 0.001), thus validating H2a. The instrumental use of social media platforms exerts a significant positive influence on work-family conflict (*β* = 0.681, *p* < 0.001), thereby validating H3a. Model 7 incorporated digital fatigue into Model 6, revealing that digital fatigue is significantly positively associated with emotional exhaustion (*β* = 0.775, *p* < 0.001). Compared to Model 6, the coefficient for instrumental use of social media on emotional exhaustion decreased from 0.667 to 0.179 in Model 7, indicating that digital fatigue partially mediates the relationship between instrumental use of social media and emotional exhaustion, thus validating H2b. Model 8 incorporated work-family conflict into Model 6, revealing a significant positive effect of work-family conflict on emotional exhaustion (*β* = 0.749, *p* < 0.001). Compared to Model 6, the effect coefficient of inclusive instrumental use of social media on emotional exhaustion decreased from 0.667 to 0.157 in Model 8, indicating that work-family conflict partially mediates the relationship between instrumental use of social media and emotional exhaustion, thus validating H3b.

**Table 5:** Results of main effects and mediating effects tests.

Variables	Digital fatigue				Work-family conflict				Emotional exhaustion							
	M1		M2		M3		M4		M5		M6		M7		M8	
	*β*	*t*	*β*	*t*	*β*	*t*	*β*	*t*	*β*	*t*	*β*	*t*	*β*	*t*	*β*	*t*
Gender	−0.038	−0.785	−0.034	−0.927	−0.060	−1.270	−0.056	−1.663	−0.043	−0.862	−0.039	−1.054	−0.012	−0.526	0.003	0.156
Age	0.145^*^	2.311	0.096^*^	1.990	0.137^*^	2.201	0.084	1.884	0.107	1.673	0.055	1.153	−0.019	−0.652	−0.008	−0.234
Education level	0.246	4.259	0.128	2.851	0.254	4.462	0.126^**^	3.087	0.254	4.317	0.129^**^	2.904	0.030	1.006	0.034	1.024
Job level	0.005	0.088	−0.053	−1.178	0.023	0.343	−0.040	−1.031	−0.062	−1.050	−0.124^**^	−2.791	−0.083^**^	−2.998	−0.092^**^	−2.891
Instrumental use of social media			0.630^***^	16.258			0.681^***^	19.000			0.667^***^	17.429	0.179^***^	5.770	0.157^***^	4.109
Digital fatigue													0.775^***^	24.420		19.007
Work-family conflict															0.749^***^	
*R* ^2^	0.125		0.487		0.136		0.560		0.092		0.498		0.807		0.745	
Adjusted *R*^2^	0.115		0.480		0.127		0.554		0.082		0.492		0.804		0.741	
Δ*R*^2^	0.125		0.362		0.136		0.424		0.092		0.406		0.308		0.246	
*F*-value	13.378		71.063		14.854		95.462		9.529		74.518		260.066		181.183	
Δ*F*	13.378		264.330		14.854		361.002		9.529		303.777		596.318		361.275	
Cohen's *f*^2^			0.706				0.964				0.809		1.601		0.969	

^*^p < 0.05; ^**^p < 0.01; ^***^p < 0.001.

N = 381.

#### 3.3.6 Testing the chain mediating effect

As shown in Table 6, Using Model 6 of the SPSS plugin PROCESS provided by Hayes (2013), instrumentalized use of social media served as the independent variable, emotional exhaustion as the dependent variable, digital fatigue and work-family conflict as chain mediating variables, and gender, age, educational attainment, and job level as control variables. The entire regression equation was significant: *R*^2^ = 0.809, *F* = 532.280, *p* < 0.001. Bootstrap sampling was employed to examine the mediating effects. Results indicated that the path indirect effect with digital fatigue as the mediating variable was 0.421 (95% CI = [0.322, 0.518]), while the path indirect effect with work-family conflict as the mediating variable was 0.055 (95% CI = [0.026, 0.090]), and the path indirect effect with digital fatigue and work-family conflict as mediating variables was 0.122 (95% CI = [0.060, 0.188]). The total indirect effects amounted to 0.598 (95% CI = [0.522, 0.674]) establishing the chained mediating role of digital fatigue and work-family conflict in the positive effect of instrumental social media use on emotional exhaustion. Hypothesis 4 is thus validated.

**Table 6:** Bootstrap chain-based mediator model test results.

Path	Effect	SE	95% CI	
Total effect	0.717	0.039	0.641	0.793
Direct effect	0.119	0.034	0.051	0.186
Indirect effect	0.598	0.039	0.522	0.674
Ind1 instrumental use of social media platforms = >digital fatigue = > emotional exhaustion	0.421	0.050	0.322	0.518
Ind2 instrumental use of social media platforms = > work-family conflict = > emotional exhaustion	0.055	0.017	0.026	0.090
Ind3 instrumental use of social media platforms = > work-family conflict = >emotional exhaustion	0.122	0.033	0.060	0.188

#### 3.3.7 Moderation effect test

As shown in Table 7, after incorporating the interaction term, the *R*^2^ value of Model 2 increased from 0.735 to 0.739, indicating improved model fit compared to the previous version. Secondly, the standardized coefficient of the interaction term is −0.064 (*P* < 0.05), indicating a significant regression result. Moreover, the regression coefficient for instrumental use of social media on digital fatigue is 0.854, which is opposite in direction to the interaction term's coefficient for digital fatigue. This indicates a significant negative moderating association, demonstrating that digital literacy exerts a negative moderating influence on the relationship between instrumental use of social media and digital fatigue. Thus, hypothesis H5 is supported.

**Table 7:** The moderating role of digital literacy in the relationship between instrumental use of social media platforms and digital fatigue.

Dependent variable: digital fatigue								
Model	Variable	Unstandardization coefficient		Standardized coefficient	*t*	Significance	*R* ^2^	Adjusted *R*^2^
		*B*	Standard Error					
1	(Constant)	2.565	0.138		18.580	0.000	0.735	0.734
	IU	0.929	0.030	0.861	30.710	0.000		
	DL	−0.730	0.037	−0.560	−19.975	0.000		
2	(Constant)	2.635	0.140		18.781	0.000	0.739	0.737
	IU	0.921	0.030	0.854	30.461	0.000		
	DL	−0.736	0.036	−0.565	−20.216	0.000		
	IU^*^DL	−0.092	0.039	−0.064	−2.387	0.017		
3	(Constant)	3.624	0.039		93.209	0.000	0.754	0.751
	IU	0.967	0.056	0.896	17.162	0.000		
	DL	−0.600	0.046	−0.461	−12.960	0.000		
	IU × DL	−0.319	0.069	−0.220	−4.638	0.000		
	IU^2^	−0.016	0.040	−0.020	−0.400	0.689		
	IU^2^×DL	−0.199	0.043	−0.253	−4.639	0.000		

IU, instrumental use of social media platforms. DL, digital literacy.

To further examine whether the moderating effect of digital literacy on the relationship between instrumental use of social media platforms and digital fatigue exhibits a nonlinear pattern, we extended the linear moderation model by incorporating the quadratic term of instrumental use and its interaction with digital literacy, thereby constructing a polynomial regression model. All continuous variables were mean-centered prior to the formation of product terms to reduce multicollinearity. The regression results, as shown in Table 7, revealed that the quadratic interaction term was significant (*β* = −0.253, *p* < 0.001), whereas the quadratic term of instrumental use alone was not (*β* = −0.020, *p* = 0.689). This indicates that the relationship between digital literacy and instrumental use is not a simple linear moderation, but rather that digital literacy influences the accumulation of digital fatigue by altering the curvature of the relationship. Moreover, compared with the model containing only the linear interaction term, the inclusion of the quadratic interaction term led to a significant increase in explanatory power (Δ*R*^2^ = 0.015, *p* < 0.001), further supporting the presence of a nonlinear moderating effect.

The simple slope analysis (Table 8) showed that the instantaneous effect of instrumental use on digital fatigue was strongest in the low digital literacy group (Slope = 1.207, SE = 0.094, *t* = 12.84, *p* < 0.001), weakened in the moderate digital literacy group (Slope = 0.967, SE = 0.055, *t* = 17.65, *p* < 0.001), and was weakest in the high digital literacy group (Slope = 0.727, SE = 0.053, *t* = 13.69, *p* < 0.001). All three slopes were significantly positive but showed a decreasing trend, indicating that higher levels of digital literacy were associated with a more gradual positive effect of instrumental use on digital fatigue, thereby further confirming the moderating role of digital literacy on the shape of the relationship.

**Table 8:** Simple slopes analysis of instrumental use on digital fatigue at different levels of digital literacy.

Digital literacy level	Slope equation	SE	*t*	*p*	95% CI
Low DL (−1SD)	Slope = 1.207 + 0.267 × IU	0.094	12.84	< 0.001	[1.022, 1.392]
Mean DL	Slope = 0.967 – 0.032	0.055	17.65	< 0.001	[0.859, 1.075]
High DL (+1SD)	Slope = 0.727 – 0.331 × IU	0.053	13.69	< 0.001	[0.622, 0.831]

IU, instrumental use of social media platforms; DL, digital literacy.

The curvature analysis (see Table 9) revealed that digital literacy significantly altered the curvilinear shape of the relationship between instrumental use and digital fatigue. Specifically, in the low digital literacy group, the curvature was significantly positive (Curvature = 0.268, SE = 0.112, *t* = 2.39, *p* = 0.017), indicating a concave-up trajectory with an accelerating increase in fatigue. In the high digital literacy group, the curvature was significantly negative (Curvature = −0.332, SE = 0.112, *t* = −2.96, *p* = 0.003), indicating a concave-down trajectory with a decelerating (diminishing) increase in fatigue. In the moderate digital literacy group, the curvature was not significant (Curvature = −0.032, *p* = 0.721), suggesting an approximately linear pattern.

**Table 9:** Curvature analysis of the relationship between instrumental use and digital fatigue at different levels of digital literacy.

Digital literacy level	Curvature	SE	*t*	*p*	95% CI
Low DL (−1SD)	0.268	0.112	2.39	0.017	[0.048, 0.488]
Mean DL	−0.032	−0.032	0.089	0.721	[−0.207, 0.143]
High DL (+1SD)	−0.332	−0.332	0.112	0.003	[−0.552, −0.112]

DL, digital literacy.

**Figure 2:**
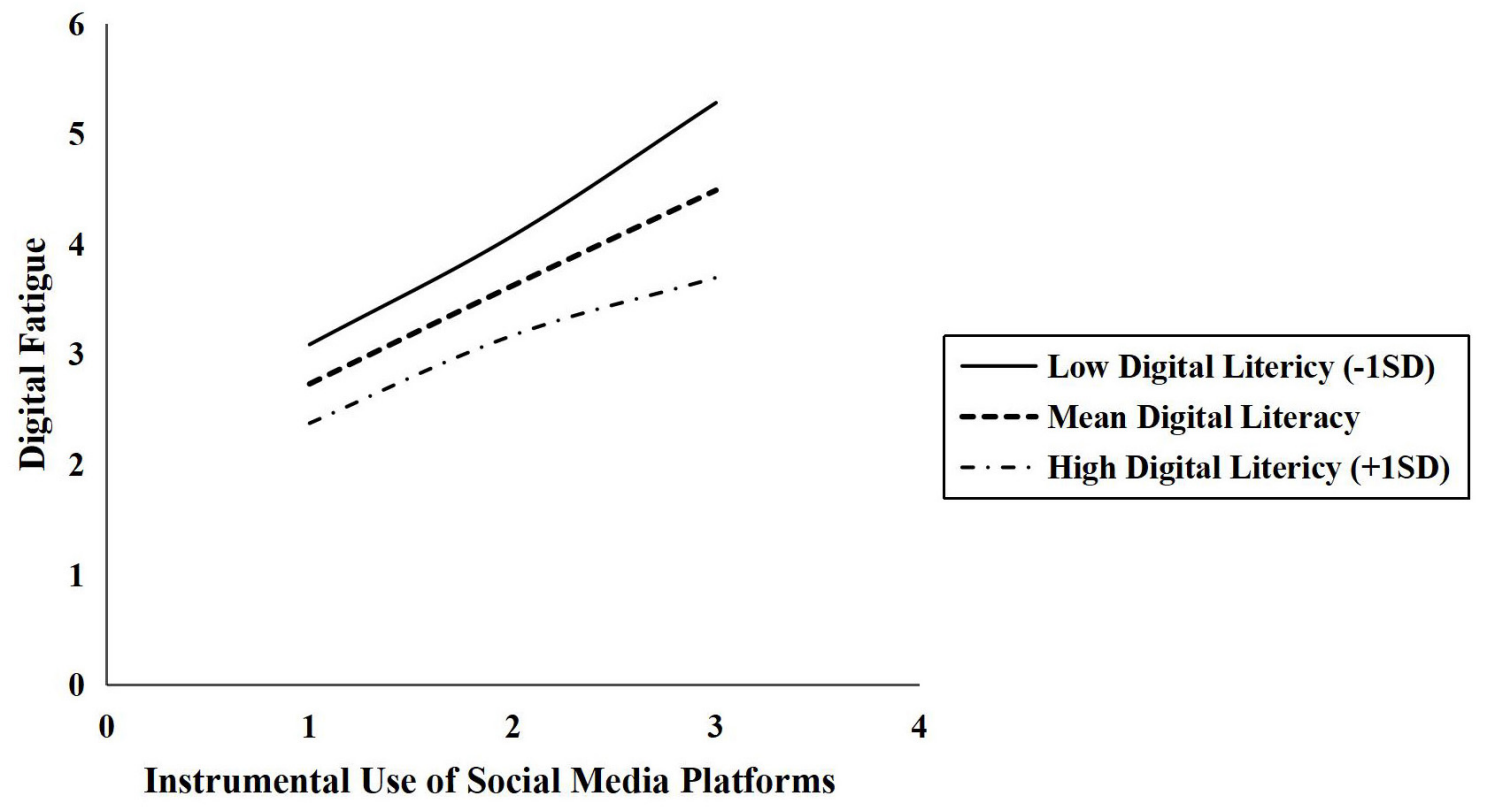
The nonlinear moderating role of digital literacy in the relationship between Instrumental use of social media platforms and digital fatigue.

To visually illustrate the nonlinear moderating effect described above, a moderation effect diagram was constructed (see Figure 2). As shown in Figure 2, the three curves represent the relationships between instrumental use and digital fatigue at low, mean, and high levels of digital literacy, respectively. In the low digital literacy group, the trajectory is concave upward, indicating that digital fatigue increases at an accelerating rate as instrumental use intensifies. In the high digital literacy group, the trajectory is concave downward, indicating that although fatigue still rises with increasing use, the rate of increase gradually decelerates, reflecting a diminishing-returns or saturating pattern. In the mean digital literacy group, the curve is approximately linear, with fatigue increasing at a nearly constant rate.

## 4 Research findings and discussion

### 4.1 Theoretical implications

This study, integrating COR theory and boundary theory, collected 381 valid samples using established scales to examine the relationship between instrumental use of social media in organizations and employees' emotional exhaustion (Ozimek et al., 2025). All hypotheses (H1–H5) were supported.

First, instrumental use of social media in organizations is positively associated with employees' emotional exhaustion. In digitalized workplaces, employees rely on social media to handle tasks and transmit information. Prolonged high-frequency and high-intensity communication can excessively deplete psychological resources, thereby exacerbating emotional exhaustion. Organizations should foster a positive interaction ecosystem and develop targeted strategies based on individual differences to alleviate emotional strain.

Second, the mediating role of digital fatigue is confirmed. Instrumental use of social media in organizations leads to employees' emotional exhaustion by increasing their digital fatigue. Prolonged exposure to and processing of fragmented, repetitive work-related information can induce a state of digital fatigue. Digital fatigue represents a severe physical and mental depletion, not only triggering aversive psychological reactions but also eliciting negative emotions and cognitive confusion (Supriyadi et al., 2025). Over time, employees' positive feelings toward the organization are gradually eroded, causing serious issues. In organizations, instrumental use of social media often leads to emotional exhaustion through digital fatigue as a mediating process. Therefore, to effectively alleviate employees' emotional exhaustion, it is essential to interrupt this chain of effects mediated by digital fatigue.

Third, the mediating role of work–family conflict is confirmed. Social media instrumental use may intensify the spillover of work demands into the family domain, thereby triggering work–family conflict and subsequently leading to the continuous depletion of employees' emotional resources. As a cumulative stress experience, the effects of work–family conflict typically emerge gradually through sustained resource depletion over time. In an increasingly digitalized work environment, even a relatively small increase in work–family conflict may accumulate over time and ultimately impair employees' emotional resources and psychological wellbeing. This finding provides a new theoretical perspective for understanding the transmission mechanisms of work-related stress in the digital era.

Fourth, the sequential mediating role of digital fatigue and work–family conflict is confirmed. Instrumental use of social media is closely associated with employees' experience of digital fatigue, and this sustained state of digital exhaustion further intensifies the encroachment of work on family life, ultimately leading to continuous depletion of emotional resources. This chain of effects reveals the full process through which stress is transmitted in a digital work environment: overuse of technological tools not only directly depletes individuals' cognitive and emotional resources but also indirectly exacerbates emotional exhaustion by disrupting the balance between work and family. The findings deepen the theoretical understanding of how digital transformation impacts employees' mental health and expand the research perspective on the relationship between digital work environments and individual psychological states (Dengler et al., 2022).

Fifth, digital literacy plays a nonlinear moderating role between instrumental use of social media platforms and digital fatigue. The study reveals that digital literacy does not merely attenuate the impact of instrumental use on digital fatigue; rather, it alters the developmental trajectory of the relationship between these two factors. Specifically, for individuals with low levels of digital literacy, as their instrumental use of social media increases, digital fatigue exhibits an accelerated cumulative trend, indicating that users lacking effective digital management skills are more susceptible to the influences of information overload, task-switching, and technical stress. Conversely, for individuals with high levels of digital literacy, a diminishing marginal effect is observed between instrumental use and digital fatigue—that is, as usage levels increase further, the rate of growth in digital fatigue gradually slows down, demonstrating the adaptive and moderating role of digital literacy in high-intensity digital environments. This finding highlights the value of digital literacy as a crucial personal resource, enriches the application of the Resource Protection Theory within digital work environments, and provides a new theoretical perspective for understanding how employees can construct self-protection mechanisms in technological-mediated environments.

### 4.2 Management implications

This study draws on Conservation of Resources theory and Boundary theory to explain the mechanisms, examining the relationship between employees' instrumental use of corporate social media and emotional exhaustion, as well as the moderating role of digital literacy and the mediating roles of digital fatigue and work–family conflict. The findings provide practical management suggestions for organizations.

First, at the institutional level, organizations should set clear “digital disconnect” periods. For example, employees could be required to turn off nonurgent group messages during fixed daily time slots, breaking the seamless connection of social media use and creating uninterrupted deep work periods. This helps reduce cognitive load caused by continuous information inflow. At the same time, technological tools should focus on filtering and simplifying information, pushing only critical content and condensing long messages into key points. The introduction of such tools is essentially aimed at combating indiscriminate “information bombardment” from social media, freeing employees from unnecessary information filtering and alleviating emotional exhaustion caused by information overload.

Second, the instrumental use of social media erodes employees' personal time by blurring the boundaries between work and life, which is a core mechanism leading to emotional exhaustion. Therefore, effective boundary management strategies should aim to help employees psychologically disconnect from work (Wang et al., 2024). Organizations should fully implement “no-disturbance after work” policies, automatically turning off notifications during nonworking h and strictly limiting nonurgent contacts. This approach not only respects employees' right to rest but also supports the recovery of their psychological resources.

Finally, organizations should include “digital fatigue” as a core indicator in monitoring and intervention systems. Regular assessments should extend to anonymized data on social media usage duration, message reply frequency, and similar metrics to quantify the impact of instrumental platform use on employees' mental state. Organizations can provide high-risk employees with customized interventions, such as “digital detox workshops” or mindfulness training (Weaver and Swank, 2024), to mitigate emotional exhaustion caused by social media's instrumental use, ensuring long-term employee wellbeing.

### 4.3 Limitations and future extensions

This study, drawing on previous literature, applies Conservation of Resources (COR) theory and boundary theory to examine how instrumental use of social media in organizations influences employees' emotional exhaustion, with digital fatigue and work–family conflict as mediators and digital literacy as a moderator.

#### 4.3.1 Common method bias

The data in this study were collected through self-reported questionnaires, which may be susceptible to social desirability bias and consequently introduce common method bias. To minimize this potential risk, several procedural remedies were implemented during the questionnaire design stage, including ensuring anonymity, reducing ambiguity in item wording, and separating predictor variables and criterion variables in the questionnaire layout. However, these procedural controls cannot completely eliminate the threat of common method bias. The observed relationships among variables may still be partially influenced by respondents' response tendencies, social desirability concerns, or their temporary emotional states. For example, employees may underestimate their own levels of exhaustion or downplay their experiences of work–family conflict in an effort to maintain a positive self-image or conform to perceived social norms, thereby affecting the accuracy of the measurements. Furthermore, this study was conducted within the specific cultural and organizational context of China. In this context, patterns of technology adoption, workplace communication norms, and employees' perceptions of work–life boundaries may differ from those in other cultural environments. Therefore, the generalizability of the findings across different countries and cultural contexts should be interpreted with caution. Future research could adopt multi-source data collection approaches, such as incorporating supervisor ratings, coworker evaluations, or objective behavioral data, and employ longitudinal tracking, time-lagged research designs, or experimental methods to strengthen causal inference and reduce the potential influence of common method bias on research conclusions. In addition, future studies could conduct cross-cultural replications by collecting data from different countries or regions to examine whether the proposed relationships remain consistent across diverse cultural and institutional environments.

#### 4.3.2 Regarding research content

Emotional exhaustion is a state that can vary depending on individuals' environment and mood (Edú-Valsania et al., 2022). The data collected in this study are cross-sectional and based on employees' self-reported questionnaires, which may limit the generalizability of the findings (Anvari et al., 2023; Wang and Cheng, 2020). Moreover, the possibility of reverse causality cannot be completely ruled out. For example, employees experiencing higher levels of emotional exhaustion may be more likely to engage in frequent social media use as a coping strategy to seek social support, regulate negative emotions, or temporarily alleviate work-related stress. Similarly, employees with higher levels of digital fatigue or work–family conflict may adjust their social media usage behaviors in response to their psychological strain. Future research could employ longitudinal designs, experience sampling methods (ESM), or experimental approaches to further clarify the causal directions among these variables and explore their dynamic relationships over time.

#### 4.3.3 Theoretical perspective

This study primarily draws on COR theory and boundary theory to construct the research framework. While these theories effectively explain the relationship between resource depletion and emotional exhaustion, they do not fully account for the moderating effects of individual differences (e.g., Big Five personality traits, psychological resilience) or organizational culture (e.g., innovation-oriented vs. rule-oriented cultures) (Spiridon et al., 2026; Lei et al., 2025). Future research could integrate Trait Activation Theory to explore how different personality traits influence employees' responses to digital tool use (Ye et al., 2024), or incorporate Situational Strength Theory to analyze how organizational cultural climate moderates the formation of digital fatigue (Supriyadi et al., 2025), thereby developing a more comprehensive theoretical framework.

#### 4.3.4 Contextual applicability

With the accelerating digital transformation, emerging technologies such as AI-based intelligent assistants and metaverse virtual work environments are reshaping work contexts (Pereira et al., 2023; Yawised et al., 2022). The impact of these technologies on the mechanisms of work-related stress was not addressed in the present study. This limitation may reduce the timeliness and applicability of the findings in rapidly evolving digital workplaces. Future research could draw on Technology Fit Theory (Zhang et al., 2025) to examine how the alignment between specific technological features and job demands affects employees' emotional exhaustion, or apply Human–Computer Interaction (HCI) theories (Kotian and Nandipi, 2024) to analyze the stress formation mechanisms in novel human–machine collaboration scenarios.

#### 4.3.5 Magnitude of the moderating effect

Although digital literacy significantly moderated the relationship between instrumental use of social media and digital fatigue, the magnitude of this effect was relatively small. This finding suggests that the buffering role of digital literacy may not be equally salient across all employees. One possible explanation is that digital literacy exerts a stronger moderating effect primarily among individuals with relatively low levels of digital competence. For these employees, prolonged online work and continuous work-related communication often require greater cognitive effort and adaptation, placing greater demands on their digital coping capacity and consequently accelerating the depletion of cognitive and emotional resources, thereby resulting in higher levels of digital fatigue. In contrast, for employees who already possess moderate or high levels of digital literacy, further improvements in digital competence may provide only limited additional benefits, resulting in a relatively small overall moderating effect.

## Data Availability

The original contributions presented in the study are included in the article/supplementary material, further inquiries can be directed to the corresponding author.
